# Disparities in Thoracic Oncology Patients

**DOI:** 10.3390/cancers18050793

**Published:** 2026-02-28

**Authors:** Mohammad W. Awlad Mohammad, Kinda Abu Hashhash, Rita Yacoub, Firas Abu Akar

**Affiliations:** 1 Faculty of Medicine, Al-Quds University, East Jerusalem 20002, Palestine; mohammadwj79@gmail.com (M.W.A.M.); kenda.abuhashhash@students.alquds.edu (K.A.H.); rita.yacoub22@gmail.com (R.Y.); 2 Department of General Surgery, Faculty of Medicine, Al-Quds University, East Jerusalem 2002, Palestine; 3 Department of Thoracic Surgery, The Edith Wolfson Medical Center, Holon 58100, Israel; 4 Sackler Faculty of Medicine, Tel Aviv University, Tel Aviv 6997801, Israel

**Keywords:** lung cancer, disparities, epidemiology treatment, screening

## Abstract

**Background/Objectives:** Lung cancer is one of the major causes of mortality worldwide despite breakthroughs in screening, diagnosis, and treatment. These advances have not been evenly spread, and discrepancies between populations remain concerning. This article examines lung cancer discrepancies in epidemiology, risk factors, screening, diagnosis, treatment access and quality, and survival outcomes, and identifies the main causes. **Methods:** An extensive narrative evaluation of peer-reviewed literature, national cancer surveillance reports, and large population-based research was searched. The evidence on lung cancer disparities by race, ethnicity, socioeconomic status, sex, geography, and healthcare access was synthesized. Disparities in prevention, early identification, treatment, and outcomes were organized into this paper. **Results:** Lung cancer incidence, stage, treatment, and survival showed persistent differences. Racial and ethnic minority groups, people on low-incomes, uninsured people, and rural or resource-limited people had higher disease burden and worse outcomes. Access to low-dose computed tomography screening, rapid diagnostic follow-up, surgical resection, molecular testing, targeted medicines, immunotherapy, palliative care, and clinical trials was unequal. When guidelines are followed, survival outcomes are similar across races and ethnicities. **Conclusions:** Lung cancer disparities are mostly caused by structural, social, and healthcare system factors, not biology. Coordinated measures to provide equitable screening, prompt and high-quality treatment, research inclusion, and culturally sensitive and policy-driven actions are needed to enhance lung cancer outcomes.

## Simple Summary

Lung cancer continues to be a predominant cause of cancer-related mortality globally; however, not all individuals experience equal advantages from advancements in screening, diagnosis, and treatment. Countless individuals from underprivileged backgrounds face elevated risks, delayed diagnoses, and worse access to adequate healthcare, resulting in adverse consequences. This review aimed to elucidate the impact of social, economic, racial, and geographic determinants on lung cancer along the continuum of care, encompassing risk exposure, early detection, treatment, and survival outcomes. The authors intend to consolidate existing research to elucidate the locations and reasons for these inequalities and their impact on patient outcomes. The results underscore that enhancing lung cancer survival necessitates not only medical advancements but also equitable access to screening, prompt diagnosis, effective treatment, and involvement in research. This study may inform future research, policy, and healthcare practices aimed at achieving equitable lung cancer care for all demographics.

## 1. Introduction

Lung (and bronchus) cancer ranks as the second most prevalent cancer diagnosed among both men and women in the United States, and it is the leading cause of cancer-related fatalities, with an estimated 127,070 deaths projected for 2023 [1,2]. It is typically lethal due to late-stage diagnoses and frequently unsuccessful treatments [2]. Additionally, strong biotech medications have emerged, aiming to enable customized treatment based on tumor biomarkers and mutation measurements. Additionally, recent low-dose computed tomography screening trials have demonstrated a notable decrease in mortality [3].

Health disparities include differences in health outcomes, such as life expectancy, mortality rates, health status, and the prevalence of health issues [4], as well as avoidable differences in health outcomes and possibilities for optimal health among socially disadvantaged communities [5]. Health care inequalities include differences between populations in terms of health insurance coverage, access to care, affordability, use of public health services, and the standard of care provided [4]. Race and ethnicity, financial class, age, region, language, gender, citizenship status, handicap status, and sexual identity and orientation are some of the elements that contribute to disparities. Some individuals encounter discrepancies across various dimensions, reflecting the intersectional character of their identities [4].

Notably, understanding the interaction of disparities within historically disadvantaged, racially disenfranchised populations is crucial for promoting social justice and reducing health inequities in the population [5].

The primary aim of this narrative review is to synthesize contemporary evidence on lung cancer disparities with a focused emphasis on race/ethnicity, socioeconomic status (SES), insurance coverage, and geography as key axes of inequity across the care continuum. Specifically, we prioritize disparities in risk exposure and prevention (including smoking, occupational, and environmental hazards), screening and early detection (such as LDCT eligibility, uptake, and follow-up), diagnosis and staging, access to molecular testing and precision oncology, treatment delivery, and clinical outcomes. Rather than cataloging all possible disparities, this review centers on mechanisms through which structural and healthcare-system factors (rather than intrinsic biological differences) drive inequitable lung cancer outcomes.

## 2. Methodology

This narrative review was conducted to synthesize current evidence on disparities in lung (and bronchus) cancer across epidemiology, risk factors, screening, diagnosis, treatment, and outcomes. A structured methodology was employed to ensure transparency, reproducibility, and comprehensiveness.

### 2.1. Literature Search Strategy

A comprehensive literature search was performed in the following electronic databases: PubMed/MEDLINE, Web of Science, Scopus, and Embase. Additional sources included the American Cancer Society (ACS) reports, National Cancer Institute (NCI) publications, World Health Organization (WHO) reports, and reference lists of relevant articles.

Search terms were constructed using Medical Subject Headings (MeSH) and keywords, including combinations of the following:

- “lung cancer” OR “bronchus cancer” OR “non-small cell lung cancer” OR “small cell lung cancer”;
- “health disparities” OR “health inequities” OR “socioeconomic status” OR “race” OR “ethnicity” OR “gender”;
- “screening” OR “diagnosis” OR “treatment” OR “mortality” OR “survival”.

Boolean operators (AND, OR) were applied to combine terms, and filters were used to select studies published in English between 2000 and 2025 to ensure contemporary relevance.

### 2.2. Inclusion and Exclusion Criteria

Articles were included if they:

- Reported on lung cancer incidence, risk factors, screening, diagnosis, treatment, or survival outcomes stratified by sociodemographic variables;
- Examined disparities by race, ethnicity, gender, socioeconomic status, geography, or insurance coverage;
- Were original research studies, systematic reviews, meta-analyses, or authoritative reports;
- Were published in peer-reviewed journals or by reputable health organizations.

Exclusion criteria were as follows:

- Studies not focused on human subjects;
- Case reports, conference abstracts, editorials, or opinion pieces without original data;
- Articles in languages other than English;
- Studies lacking relevant demographic or disparity-specific analyses.

### 2.3. Data Synthesis and Organization

Selected evidence was synthesized qualitatively, using a narrative approach. Studies were organized into thematic domains: epidemiology, risk factors, screening, diagnosis and staging, treatment access and quality, and clinical outcomes. Patterns of disparities across race, ethnicity, SES, gender, and geography were highlighted. The narrative approach allowed integration of quantitative and qualitative findings and facilitated discussion of contextual factors, policy implications, and intervention strategies.

### 2.4. Scope and Limitations

This review is a narrative synthesis rather than a systematic review or meta-analysis. While it draws on a broad range of contemporary literature, it does not quantitatively pool data or formally assess study quality. The narrative approach provides a comprehensive overview of disparities and trends but may be susceptible to selection bias and publication bias. Additionally, variations in study populations, definitions of socioeconomic status, and measurement of outcomes limit the generalizability of some findings.

## 3. Epidemiology and Disparities in Lung Cancer

The number of new lung cancer cases in 2025 was estimated to be 226,650, representing 11.1% of all new cancer cases; estimated deaths in 2025 were 124,730, i.e., 20.2% of all cancer deaths, with a 5-year relative survival rate of 28.1% within 2015–2021 [6]. According to the American Cancer Society’s 2025 report, overall cancer incidence has declined in men but risen in women, leading to a narrowed-incidence male-to-female ratio. Crucially, the NCI’s 2025 report notes that lung (and bronchus) cancer incidence and mortality have continued to decline, particularly driven by reductions in smoking, a primary risk factor [2].

A variety of neoplasms with distinct biological characteristics make up lung cancer [7]. The two main forms of lung cancer include Small cell lung cancer (SCLC), which makes up 14% of cases, and non-small cell lung cancer (NSCLC), which makes up 81% of cases [3]. NSCLC is further divided into squamous cell carcinoma and large cell carcinoma, with adenocarcinoma being slightly more frequent in women (17.1%) [8].

Cancer ranks as the second leading cause of mortality in the United States and is the primary cause among individuals under 85 years of age [9]. Lung (and bronchus) cancer is the leading cause of cancer-related fatalities [1,2,3]. Lung cancer constitutes the most prevalent cancer diagnosed globally (excluding keratinocyte carcinoma) and is the leading cause of cancer-related mortality. It comprises around 17% and 9% of all cancers in males and females, respectively, and accounts for 19% of all cancer-related fatalities [2].

The American Cancer Society estimates the prevalence of lung cancer in the US in 2025 to be about 226,650 new cases of lung cancer (110,680 in men and 115,970 in women) and about 124,730 deaths (64,190 in men and 60,540 in women) [10].

Notably, many factors determine the incidence rate of lung cancer, including gender, race/ethnicity, socioeconomic level, and region, as shown in Table 1. Some studies hypothesize that these differences are primarily due to variations in cigarette smoking behaviors [11]. Black males, those with lower socioeconomic positions, and those residing in the South have a greater prevalence of lung cancer [12].

From an epidemiological perspective, the National Cancer Institute defines cancer disparities as harmful differences in cancer incidence, prevalence, mortality, survivorship, and burden among specific demographic groups [12].

**Figure 1:**
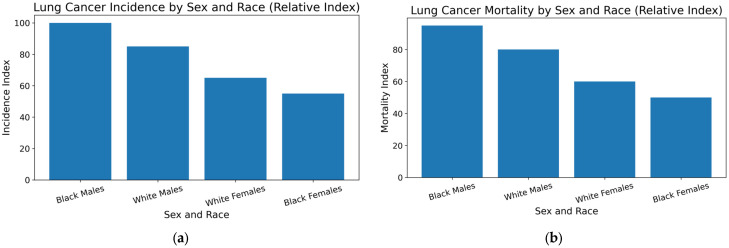
Lung cancer incidence (**a**) and mortality (**b**) by sex and race in the United States. Data source: National Cancer Institute SEER Program; years: 2015–2021; rates age-adjusted to the 2000 U.S. standard population.

There is a clear disparity in the incidence of mortality and morbidity between white and Black individuals, as shown in Figure 1. Between 1999 and 2012, a longitudinal study assessed differences in lung cancer rates between Black and White males and females in 38 U.S. states, the District of Columbia, and eight U.S. geographic regions. Using a longitudinal linear mixed-effects model, they demonstrated that although racial and gender disparities in lung cancer incidence persist, age-adjusted incidence rates have decreased across the United States. Black people continue to have higher age-adjusted incidence rates of lung cancer than white people, even if the racial disparity has decreased over time. These differences are more noticeable in males than in women. Among all demographic subgroups, Black males had the greatest lung cancer prevalence, followed by White males, White females, and lastly Black females [13].

Males continue to have substantially higher rates of cigarette smoking than females at all time points, which is mirrored in their higher incidence rates of lung cancer at all time points, despite a clear downward trend in cigarette smoking in both genders [13,14,15]. But even while cigarette smoking has clearly decreased for both sexes, men continue to smoke significantly more than women at all times, as seen by their higher prevalence of lung cancer during [15,16,17].

Nearly half (49%) of all lung cancer cases currently arise in nations classified as medium to poor on the Human Development Index (HDI) [16].

Countries showed marked differences in sex-specific lung cancer incidence rates. Among males, the estimated incidence ranged from fewer than 5 per 100,000 population in several African countries (e.g., Kenya) to as high as 104 per 100,000 in Hungary. In contrast, the highest estimated incidence among females was reported in the United States (40 per 100,000), while much lower rates were observed in less developed countries such as India, Algeria, Kenya, and Iran, where incidence ranged between 2 and 3 per 100,000 population [17].

People with a lower socioeconomic status (SES) were more likely to develop lung cancer. The mortality rate from lung cancer is almost five times greater for men and four times higher for women aged 25–74 with less than 12 years of education than it is for those with at least 16 years of education [3]. Compared to 3% of people with a doctorate degree, 21% of those without a high school education and 31% of people with a GED smoked cigarettes in 2021 [3].

Across epidemiologic studies, racial differences in lung cancer incidence are closely aligned with historical and contextual smoking patterns, targeted tobacco marketing, occupational exposures, and neighborhood-level deprivation. Importantly, while incidence disparities have narrowed over time, mortality disparities persisted, suggesting that downstream factors such as delayed diagnosis, treatment access, and quality of care play a dominant role. Sex-based differences largely mirror smoking prevalence; however, women demonstrate superior survival, indicating possible interactions between biology, health-seeking behavior, and care delivery. Table 2 summarizes major lung cancer disparities across the care continuum by sociodemographic axis, clinical metric, and outcome direction.

## 4. Disparities in Risk Factors and Screening

There are numerous behavioral, biological, environmental, social, and demographic factors that influence lung cancer risk. Smoking remains the single most important risk factor; however, a growing body of research demonstrates how differences in race, income, and access to healthcare affect the rates and outcomes of many diseases. Studies showed that people of lower socioeconomic status had lower screening rates and later detection, in addition to smoking behavior and other health problems. One potential reason is that Socioeconomic factors that affect health, such as structural racism, limited access to healthcare, and living in poverty, have historically influenced people’s exposure to risk, access to preventive treatments, and the speed of their diagnosis. People with mental health or substance use disorders are at a greater risk because they are less likely to be screened regularly, meaning they are more likely to have advanced disease at diagnosis. In addition, being older than 70, never married, and currently a smoker are all characteristics associated with lower screening adherence. These factors are considered multipliers of clinical and social risk. These differences are important because they often remain the same even after adjusting for multiple variables, i.e., there may be hidden or systematic biases that are not taken into account [18,19]. Additionally, some habits are linked to an increased risk of lung cancer. Research data reveal that a diet characterized by high intake of fruits, vegetables, breakfast cereals, and dietary fiber, as well as low intake of red meat and processed meat, was associated with a lower risk of lung cancer [20,21].

It is worth noting that these trends are mostly based on large-scale studies of non-small cell lung cancer (NSCLC), which accounts for more than 85% of all lung cancer cases, and may not fully apply to small cell lung cancer (SCLC). On the other hand, more recent research reveals that Black and Latino patients with advanced NSCLC may have the same or higher chances of living than non-Hispanic white patients. This distinction reveals that NSCLC may have different risk patterns and outcomes, even though there are well-known disparities in how lung cancer is treated in different ethnic groups. This could be due to how effectively the treatment works, how easy it is to acquire both chemotherapy and radiation, or being referred to a center that specializes in treating these types of conditions. Thus, basic risk metrics can help in figuring out how risky a given group of people is, but it is also crucial to think about the type of cancer, the stage of the disease, and the treatment options that are available for lung cancer [22].

To discuss specific risk factors in detail:

### 4.1. Smoking Status

Even though tobacco use has been declining over the past few decades, it is still the cause of most lung cancer cases. This makes smoking status a very important risk factor on its own and in combination with other factors. The studies that were looked at demonstrate that current smokers are less likely to get screened for lung cancer, even if they meet the criteria set by health experts. This surprising finding highlights a major flaw in preventive engagement: current smokers who would benefit the most from early detection often have the hardest time getting involved. Some possible reasons are fatalistic beliefs, being judged in healthcare settings, and not being as involved in healthcare. In adjusted analyses, people who smoke now have a much lower chance of finishing lung cancer screening. Also, the effects of smoking are affected by other determinants, such as race and age. For example, older Black veterans who smoked showed very low screening adherence, even though they were in a high-risk group for lung cancer death. These patterns go against the idea that smoking alone explains differences in outcomes and show how important it is to have interventions that not only help people quit smoking but also make it easier for them to get care by lowering structural and psychological barriers [22]. It is important to remember that smoking is a risk factor that changes depending on socioeconomic status (SES). A study found that people in lower socioeconomic groups who smoke have a higher absolute risk of death. This means that these groups would have more public health benefits if they stopped smoking or never started [23]. It is worth noting that there is a higher risk associated with smoking compared to vaping; a systematic review indicates a potential link between electronic cigarette use and a higher risk of lung cancer, especially among individuals who use both e-cigarettes and traditional cigarettes [24]. Although current evidence is insufficient to prove causality due to varied study designs and limited long-term data, the findings suggest significant pulmonary risks associated with e-cigarette use [25].

Studies look at the relative risk of smoking among different socioeconomic groups, but this may hide differences in absolute risk because people with lower socioeconomic status are more likely to get sick from smoking. There are three more concerns with the available literature.

First, most studies only look at simple measures of smoking exposure, like whether someone is a current, ex-, or never-smoker. This does not take into account the differences in total smoking history between people of different socioeconomic backgrounds [24,25]. For instance, people with low incomes who smoke start at a younger age and smoke more cigarettes each day. Second, most studies look at general health outcomes such as all-cause mortality, meaning that the interaction between smoking and socioeconomic status may be confounded by other risk factors for general poor health [25]. Third, many studies use cross-sectional designs, and the results may be biased by selection if some non-smokers quit due to worsening health [25]. Worth noting, one cross-sectional study observed that the difference in health between smokers and non-smokers was greater for those in high-status occupations than among manual workers. The author suggested that the effects of multiple risk factors in low socioeconomic groups do not always accumulate, limiting the risks specifically attributable to smoking [25].

This is now commonly known as the Blaxter hypothesis [25], which is also known as the social vulnerability hypothesis. It proposes that unhealthy behaviors have a less detrimental impact on health for individuals in lower socioeconomic groups compared to those in higher socioeconomic groups. Individuals in lower socioeconomic groups are already exposed to numerous health risks and stressors, such as substandard housing, hazardous work environments, and poor neighborhood conditions. Therefore, the added impact of unhealthy behaviors may be less pronounced in this group [25].

Although smoking remains the primary biological risk factor, disparities in lung cancer risk are exacerbated by structural determinants such as poverty, inadequate housing, rigid employment conditions, and restricted access to preventive healthcare. Evidence consistently demonstrates that, even after adjusting for smoking intensity, individuals of lower socioeconomic status and ethnic minorities continue to show higher lung cancer mortality rates, highlighting the insufficiency of behavior-based explanations alone [22,23].

### 4.2. Occupational Carcinogens

Occupational exposures represent another significant contributor to lung cancer disparities. A systematic review confirmed asbestos and silica exposure as independent risk factors for lung cancer, even after adjustment for smoking history [26]. Cohort studies have demonstrated increased lung cancer incidence among seafarers compared with the general population, with elevated cancer risk observed in both men and women. Although higher historical tobacco and alcohol use were documented among male seafarers, these factors alone did not account for the observed excess cancer incidence [27].

Benzene exposure is also strongly associated with increased lung cancer risk, including among non-smokers. While benzene use is regulated in many regions, exposure remains prevalent in unregulated workplaces and in countries with low socioeconomic development. Workers in low-SES environments are disproportionately exposed, further amplifying cancer risk disparities [26].

### 4.3. Socioeconomic Status

Despite major advances in lung cancer diagnosis and treatment over the past two decades, individuals from lower SES groups continue to experience disproportionately worse outcomes. In the United States, lower SES is associated with reduced access to lung cancer screening and underutilization of newer treatment modalities. Studies examining SES-related disparities vary widely in how SES is defined, measured, and analyzed, with some relying on individual-level indicators and others using area-based indices.

Area-based SES measures often reveal greater survival disparities between the highest and lowest SES groups than individual-level measures. However, these findings may not be generalizable to low-income or resource-limited countries. Overall, evidence consistently demonstrates higher lung cancer risk and worse survival outcomes among individuals from socioeconomically disadvantaged backgrounds [28,29].

## 5. Race, Ethnicity, and Gender Disparities

A substantial body of literature documents persistent and significant disparities affecting racial and ethnic minority populations across the lung cancer care continuum [30,31]. Black patients, in particular, experience earlier age of onset, lower screening eligibility, higher likelihood of advanced-stage presentation, and reduced access to guideline-concordant care. After diagnosis, Black patients are less likely to undergo genetic mutation testing, surgical resection, or receive high-cost systemic therapies associated with improved survival [32].

Black patients encounter earlier disease onset, decreased eligibility for screening, lower rates of surgical resection, molecular testing, and access to high-cost systemic therapies. Comparable patterns are evident among Latino, American Indian/Alaska Native, and certain Asian American populations [30,32].

Crucially, numerous studies show that when guideline-adherent care is provided, especially within integrated systems such as the Veterans Health Administration, survival outcomes become comparable across racial and ethnic groups. These findings strongly indicate that disparities are primarily influenced by insurance status, referral practices, provider bias, geographic accessibility, and systemic racism, rather than by inherent biological differences in tumor behavior [22,30,32].

Similar disparities have been observed across other cancer types, including breast, colorectal, prostate, and hepatocellular cancers [32]. The American Lung Association’s Racial and Ethnic Disparities Report documented delayed diagnosis and reduced treatment rates among Latino, Asian American and Pacific Islander, and American Indian and Alaska Native populations compared with White patients [32].

In immunotherapy studies, African American patients were less likely to receive treatment and had lower SES scores compared with White patients, despite similar therapeutic responsiveness. These disparities reflect broader inequities in access, referral patterns, and healthcare infrastructure [30].

## 6. Access to Low-Dose CT Screening

Low-dose computed tomography (LDCT) was first proposed nearly three decades ago as a screening modality for lung cancer. Early observational studies demonstrated superior detection of early-stage malignancies compared with chest radiography. LDCT delivers lower radiation doses and is optimized for lung cancer screening [31,33].

The National Lung Screening Trial demonstrated a 20% reduction in lung cancer mortality among individuals screened with LDCT compared with chest radiography, marking a major milestone in cancer prevention [30,31]. Since then, LDCT screening has been implemented in several high-income countries, primarily through randomized controlled trials and pilot programs.

Screening programs in North America and Europe have focused predominantly on high-risk smokers, whereas studies in East Asia have included broader populations, including never-smokers. Despite proven benefits, access to LDCT screening remains uneven, particularly among racial minorities and low-income populations [31,33].

## 7. Follow-Up Disparities

Timely follow-up after abnormal lung cancer screening findings is essential for early intervention and survival. However, disparities in follow-up completion are well documented. Black veterans, for example, are significantly less likely than White veterans to complete LDCT follow-up within recommended timeframes, even after adjustment for confounders [33,34].

These gaps reflect deeper structural issues, including medical mistrust, competing life demands, and insufficient social support. Historical discrimination has fostered stigma and fatalism, undermining engagement with preventive care and smoking cessation initiatives. Addressing community perceptions and improving culturally sensitive communication are critical for effective screening implementation [35].

## 8. Education and Awareness Gaps

Educational attainment plays a critical role in lung cancer outcomes. Lower education levels are associated with increased mortality, delayed diagnosis, and reduced screening participation. Factors such as anxiety, stigma, and fatalistic beliefs disproportionately affect individuals from low-income and low-education backgrounds, particularly among current smokers [36].

Higher levels of fatalism have been associated with advanced-stage cancer diagnoses, independent of demographic and clinical factors. This association highlights the importance of targeted education and community-based interventions to improve cancer awareness and screening uptake [37].

## 9. Disparities in Diagnosis and Staging

Barriers to early lung cancer diagnosis persist globally, particularly in low- and middle-income countries. Cultural norms, environmental hazards, and inadequate tobacco control programs exacerbate disease burden. Psychological barriers, including fear, shame, and logistical challenges, further delay help-seeking behaviors among high-risk individuals [37,38,39].

Racial and socioeconomic minorities are still underrepresented in lung cancer clinical trials, limiting the generalizability of trial findings and delaying access to new medicines. Restrictive eligibility criteria, geographic distance, insufficient insurance coverage for ancillary charges, and limited provider referrals are all barriers.

Despite advances in tumor biology and precision medicine, lung cancer mortality remains high worldwide. Improved utilization of genomic and molecular diagnostics offers promise for earlier detection and personalized treatment strategies [40,41].

## 10. Precision Medicine and Biomarker Testing Disparities

Precision oncology, which includes testing for EGFR, ALK, ROS1, BRAF, KRAS, MET, RET changes, PD-L1 expression, and broad next-generation sequencing (NGS), has fundamentally revolutionized the management of non-small cell lung cancer (NSCLC). However, access to molecular diagnostics and tailored therapy is still inconsistent. Several studies have found that Black patients, uninsured or Medicaid-insured persons, and those treated in low-resource or community settings are much less likely to undergo full genetic profiling. Insurance-related restrictions, prior authorization requirements, insufficient pathology infrastructure, and delayed tissue acquisition all contribute to decreased testing rates [42,43].

Disparities in biomarker testing lead directly to inequities in therapy choices. Patients who do not undergo timely NGS are less likely to receive targeted treatments or immunotherapy, both of which have significant survival benefits. These disparities remain even in academic centers, emphasizing systemic rather than institutional explanations.

## 11. Disparities in Treatment Access and Quality

Significant disparities exist in access to surgical intervention, systemic therapy, and radiation treatment. Black men are less likely to undergo surgical resection for early-stage NSCLC compared with White men, even after surgical consultation. Physician-level variation suggests provider contributions to observed disparities [42,44].

Veterans receiving care within the Veterans Health Administration demonstrate improved outcomes despite socioeconomic deprivation, suggesting that integrated healthcare models may mitigate disparities [45]. Importantly, when Black patients receive equivalent treatment, survival outcomes are comparable to those of White patients, reinforcing the necessity of equitable care delivery [46].

## 12. Factors Contributing to Disparities

A comprehensive literature review indicated that various patient, healthcare system, and disease-related factors contribute to delays in the early identification and diagnosis of lung cancer [47]. Distrust, shame, and the fear of accountability significantly influence help-seeking behaviors, indicating the presence of both provider/system-level and patient-level barriers [47]. An example of this is the relationship between patients and general practitioners (GPs). A significant barrier to care was access, encompassing logistical and systemic issues, including appointment availability and geographical distance, alongside patient-related challenges such as socioeconomic status and knowledge gaps [47]. The lack of awareness regarding lung cancer symptoms and treatments has impacted patients, general practitioners, and the public, affecting all three domains: patient, provider/system, and disease [47]. The findings indicate that cultural sensitivity is crucial in cancer care, exemplified by the situation in Australia, where insufficient cultural competence among general practitioners has been shown to impede early diagnosis, particularly for patients from diverse backgrounds [47,48]. Delays at the system level, such as longer wait times for appointments, referrals, and diagnostic tests, as well as the cost and availability of healthcare, made it harder for patients to get treatment on time and were more difficult for patients with low socioeconomic status [47]. Insurance status also turned out to be an important factor affecting results. Research done in China showed that NSCLC patients with better insurance had better long-term survival, with benefits lasting for more than ten years. This shows how important it is to do further research on this link at the population level [48].

## 13. Efforts to Reduce Disparities

Age, gender, race/ethnicity, region, and socioeconomic status impact disparities in lung cancer screening and biomarker testing. Rapid detection of non-small-cell lung cancer (NSCLC) is critical to guide treatment. This is particularly true in underserved areas where new diagnostic tools, such as tissue and fluid biopsies, are available. comprehensive strategy is required to reduce these disparities. This means ensuring everyone has access to personalized screening and treatment, reaching out to more community members, enrolling more of them in clinical trials, and making genetic testing more affordable. To make lung cancer treatment more inclusive and effective, we need to study the socioeconomic factors that influence health, develop programs to better help people quit smoking, and provide more funding for initiatives that promote health equity [49,50].

## 14. Outcomes, Future Directions, and Recommendations

Despite improvements in lung cancer management, the overall prognosis remains poor. Survival varies by sex, race, SES, geography, and access to care. Females consistently demonstrate superior survival compared with males, and rural populations experience worse outcomes due to healthcare access limitations [51,52,53].

Action-oriented proposals include expanding LDCT eligibility criteria to better capture high-risk populations underrepresented by pack-year thresholds and implementing patient navigation and community health worker programs targeting screening follow-up and treatment initiation. Tie reimbursement and accreditation to equity metrics, including molecular testing rates and time-to-treatment. Invest in safety nets and rural oncology centers through value-based funding models. Mandate diversity benchmarks and decentralized trial designs to improve clinical trial access.

This is part of future efforts that prioritize equitable screening, universal access to molecular testing, inclusive clinical trial enrollment, and culturally competent care delivery. Precision medicine should not exacerbate existing inequities. Instead, targeted policy reform, investment in underserved communities, and global tobacco control enforcement are essential to reducing lung cancer disparities worldwide [49,50,51,52,53,54,55,56,57].

## 15. Conclusions

This review highlights the persistent and multifaceted disparities in lung cancer across the continuum of care, from risk exposure and screening to diagnosis, treatment, and survival outcomes. Disparities in lung cancer outcomes are mostly caused by structural and healthcare system issues, rather than biological variations. Addressing these disparities necessitates deliberate policy, financing, and clinical reforms to ensure that developments in thoracic oncology benefit all populations equally. Sociodemographic factors—including race, ethnicity, socioeconomic status, gender, geography, and insurance coverage—intersect to shape unequal access to preventive services, stage-appropriate treatment, and participation in clinical trials. These inequities translate into measurable differences in survival and quality of life, disproportionately burdening historically marginalized and resource-limited populations. Importantly, the evidence demonstrates that disparities are not solely driven by biological or behavioral risk factors such as smoking but are profoundly influenced by systemic barriers, including structural racism, healthcare access, provider bias, and policy shortcomings. While advances in screening technologies, molecular diagnostics, and targeted therapies hold promise for improved outcomes, their benefits remain unevenly distributed, threatening to widen existing inequities if not accompanied by deliberate equity-focused interventions.

Addressing thoracic disparities requires a multipronged approach: expanding equitable access to early detection and guideline-concordant treatment, ensuring affordability of precision medicine, diversifying clinical trial participation, and embedding culturally sensitive patient navigation strategies into health systems. Strengthened public health policies—particularly tobacco control, occupational safety, and environmental regulation—must be coupled with community-based education and advocacy to reduce risk exposures and foster trust in healthcare systems. Ultimately, reducing disparities in lung cancer is both a scientific and ethical imperative. By prioritizing equity in research, policy, and clinical practice, the oncology community can move closer to a future where advances in thoracic oncology are accessible to all patients, irrespective of their background, and where survival gains are shared equitably across populations.

## Figures and Tables

**Table 1:** Epidemiologic disparities in lung cancer by demographic and socioeconomic factors. Definitions: SES = socioeconomic status; HDI = Human Development Index.

Factor	Disparity Observed
Sex	Higher incidence and mortality in males; females show better survival
Race/Ethnicity	Higher incidence and mortality among Black males; disparities persist despite overall decline
Socioeconomic status	Higher incidence, mortality, and advanced-stage diagnosis in lower SES groups
Education level	Lung cancer mortality up to 5× higher in individuals with <12 years of education
Geographic region [U.S.]	Higher incidence in the Southern United States
Global development level	~49% of lung cancer cases occur in medium-to-low Human Development Index countrieslung cancer cases occur in medium-to-low Human Development Index
Smoking prevalence	Higher among males and individuals with lower education and SES
Urban vs. rural residence	Rural populations show poorer survival and later-stage diagnosis
Age	Lung cancer remains the leading cause of cancer death under age 85

**Table 2:** Key lung cancer disparities across the care continuum.

Disparity Axis	Care Metric	Direction of Disparity	Approximate Magnitude
**Race (Black vs. White)**	Incidence	Higher	+10–20%
**Race (Black vs. White)**	Surgical resection	Lower	−15–25%
**SES (Low vs. High)**	LDCT screening	Lower	−30–50%
**Insurance(Uninsured)**	Molecular testing	Lower	−20–40%
**Geography (Rural vs. Urban)**	Survival	Worse	HR 1.1–1.3

## Data Availability

No new data were created or analyzed in this study.

## Abbreviations

The following abbreviations are used in this manuscript:

NSCLC	non-small cell cancer
HDI	High developmental index
SES	socioeconomic status
LDCT	Low-dose computed tomography
